# Correction to " Bhatia K, Columb M, Narayan B, Wilson A; Group of Obstetric Anesthetists of Lancashire; Greater Manchester and Mersey (GOAL‐GM) Study Collaborators. Critical care, maternal and neonatal outcomes of pregnant women with COVID‐19 admitted to eight intensive care units during the wildtype, alpha and delta waves of the pandemic across the North West of England‐a retrospective review. Acta Obstet Gynecol Scand. 2023 Dec;102(12):1719‐1729. doi: 10.1111/aogs.14681. Epub 2023 Sep 20. PMID: 37727968; PMCID: PMC10619604."

**DOI:** 10.1111/aogs.14824

**Published:** 2024-03-14

The authors wish to correct a statement in the GOAL GM study investigating the critical care, maternal and neonatal outcomes of women admitted to eight intensive care units across the North West of England. The following sentence on page 1726 in the Discussion section “In the OB‐COVICU study, in patients who had expedited delivery following ICU admission, maternal outcomes deteriorated following delivery“ should read as “In the OB‐COVICU study, expedited delivery in critical patients (88% of whom needed IPPV) following ICU admission, maternal outcomes deteriorated following delivery whereas patients in the non‐critical group (14% of whom needed IPPV) were observed to improve rapidly after delivery”.^1^

The authors wish to apologise for the error.

## References

[1] Fatnic E , Blanco NL , Cobiletchi R , et al. Outcome predictors and patient progress following delivery in pregnant and postpartum patients with severe COVID‐19 pneumonitis in intensive care units in Israel (OB‐COVICU): a nationwide cohort study. Lancet Respir Med. 2023;11:520‐529. 36746165 10.1016/S2213-2600(22)00491-X PMC9949483

